# Myocilin Gene Mutation Induced Autophagy Activation Causes Dysfunction of Trabecular Meshwork Cells

**DOI:** 10.3389/fcell.2022.900777

**Published:** 2022-05-09

**Authors:** Xuejing Yan, Shen Wu, Qian Liu, Ying Cheng, Jingxue Zhang, Ningli Wang

**Affiliations:** ^1^ Beijing Tongren Eye Center, Beijing Ophthalmology and Visual Sciences Key Laboratory, Beijing Tongren Hospital, Beijing Institute of Ophthalmology, Capital Medical University, Beijing, China; ^2^ Collaborative Innovation Center for Brain Disorders, Beijing Institute of Brain Disorders, Capital Medical University, Beijing, China

**Keywords:** POAG, myocilin, trabecular meshwork, autophagy, ER stress

## Abstract

Trabecular meshwork dysfunction is the main cause of primary open angle glaucoma (POAG) associated with elevated intraocular pressure (IOP). Mutant myocilin causes glaucoma mainly via elevating IOP. Previously we have found that accumulated Asn 450 Tyr (N450Y) mutant myocilin impairs human trabecular meshwork (TM) cells by inducing chronic endoplasmic reticulum (ER) stress response *in vitro*. However, it is unclear how ER stress leads to TM damage and whether N450Y myocilin mutation is associated with POAG *in vivo*. Here we found that N450Y mutant myocilin induces autophagy, which worsens cell viability, whereas inhibition of autophagy increases viability and decreases cell death in human TM cells. Furthermore, we construct a transgenic mouse model of N450Y myocilin mutation (Tg-MYOC^N450Y^) and Tg-MYOC^N450Y^ mice exhibiting glaucoma phenotypes: IOP elevation, retinal ganglion cell loss and visual impairment. Consistent with our published *in vitro* studies, mutant myocilin fails to secrete into aqueous humor, causes ER stress and actives autophagy in Tg-MYOC^N450Y^ mice, and aqueous humor dynamics are altered in Tg-MYOC^N450Y^ mice. In summary, our studies demonstrate that activation of autophagy is correlated with pathogenesis of POAG.

## Introduction

Glaucoma is the leading cause of irreversible blindness and predicted to affect 112 million people globally by 2040 (Tham et al., 2014). Primary open angle glaucoma (POAG) is the most common type of glaucoma, characterized by progressive retinal ganglion cell (RGC) loss and visual impairment (Quigley, 1999; Weinreb et al., 2014). Intraocular pressure (IOP) elevation is the major risk factor and reducing IOP is the only treatable strategy for POAG (Rosenthal and Leske, 1980). The trabecular meshwork (TM) primarily functions in regulation of aqueous humor balance to maintain normal IOP. Dysfunction and loss of TM may impede aqueous humor outflow, thus causing IOP elevation. In POAG, cellularity reduction and architectural alteration of TM have been observed, and are associated with aqueous humor outflow reduction (Tripathi, 1972; Rodrigues et al., 1976; Alvarado et al., 1984). However, the detailed molecular mechanisms of TM injury have not been elucidated.

Autosomal dominant inherited myocilin mutations reportedly cause 2–4% of POAG and most cases of juvenile onset open-angle glaucoma (Sheffield et al., 1993; Stone et al., 1997; Kwon et al., 2009). The emerging role of wild type (WT) myocilin has been implicated in cell migration (Kwon and Tomarev, 2011), osteogenic differentiation (Kwon et al., 2013b), myelination (Kwon et al., 2013a), cell proliferation, survival and differentiation (Joe et al., 2014; Zhang et al., 2021) in non-ocular disease, but no clear consensus has been reached. Numerous studies using transgenic mouse models have demonstrated that WT myocilin is not necessary for IOP regulation (Kim et al., 2001; Wiggs and Vollrath, 2001; Zhou et al., 2008; McDowell et al., 2012). We and others have implicated that mutant myocilin protein aggregation induces ER stress and leads to death of TM cells (Jacobson et al., 2001; Joe et al., 2003; Zode et al., 2015; Yan et al., 2020). However it is unclear how N450Y mutant myocilin (MYOC)-induced ER stress impairs TM cells and whether N450Y mutant MYOC proteins are associated with the development of glaucoma *in vivo*.

Various physical and pathological stimulation could induce ER stress by disturbing homeostasis in the ER, such as a change in Ca2+ levels, aberrant post-translational modification, mutant or misfolded protein accumulation (Sun and Brodsky, 2019; Bhardwaj et al., 2020). Initially, unfolded protein response is activated to alleviate ER stress and restore ER function (Hetz, 2012; Rashid et al., 2015). but constant ER stress can stimulate autophagy (28). Autophagy is a fundamental cellular metabolic process that promotes cellular homeostasis, differentiation, development, and survival (Leidal et al., 2018). Autophagy involves sequestration of double membrane vesicle engulfed cargo called autophagosomes which subsequently fuse with lysosomes to degrade the cargo. Generally, autophagy consists of several consecutive steps: initiation, nucleation, elongation, maturation and fusion with lysosome for degradation. ULK1 kinase complex is essential for initiation of autophagy. The ULK1 complex activates Beclin1 (BECN1) dependent phosphoinositide 3-kinase (PI3K) complex for progression of the autophagy process (Egan et al., 2011). Microtubule-associated protein light chain 3 (LC3) plays a vital role in autophagosome formation, and conversion of LC3B-I to LC3B-II is an index of autophagosome formation (Huang et al., 2015). LC3B-Ⅱ binds to autophagy receptor p62 targeted for degradation. Autophagy participates in clearing accumulated mutant proteins and misfolded proteins to prevent toxic aggregation (Perlmutter, 2006; Rubinsztein, 2006; Rashid et al., 2015bib_Rashid_et_al_2015). Chronic ER stress makes a cytoprotective contribution to autophagy (Ogata et al., 2006; Ding et al., 2007). Hyperactivated autophagy may lead to cell dysfunction in metabolic and other diseases (Yamamuro et al., 2020), including glaucomatous optic neuropathy (Park et al., 2012; Park et al., 2018; Adornetto et al., 2020), however it is not understood whether N450Y mutant MYOC activates autophagy and leads to autophagy related cell death.

Turnover of intracellular WT MYOC proteins is involved in ubiquitin-proteasome and lysosomal pathways. In the case of MYOC mutation, including Q368X and P370L, ubiquitin-proteasome pathway is impaired and autophagy is induced in short term experimental observation (Qiu et al., 2014), but the role autophagy plays in the MYOC mutant cellular context is unclear. Here, we developed a transgenic mouse model of N450Y MYOC mutation (Tg-MYOC^N450Y^), to investigate whether MYOC-N450Y mutation induced constant ER stress activates autophagy and whether autophagy inhibition rescues TM cell death.

## Materials and Methods

### Cell Culture and Characterization

Two strains of primary TM cells from two individuals were purchased from the ScienCell corporation (ScienCell, Carlsbad, CA, United States; Catalog:16873; 7278). The cells were maintained at 37°C with 5% CO2 in TMCM medium (ScienCell), supplemented with 2% fetal bovine serum (ScienCell), 1% penicillin/streptomycin solution (ScienCell) and 1% TM cell growth supplement (ScienCell).

Human TM cells used in this study at passage 3-6 were treated with 100 nM dexamethasone (Selleck) for seven days and immunofluorescence staining of TM biomarkers (TIMP3, LAMA4) was applied to validate the specific origin of TM tissue.

### Lentivirus Infection

1.0*10^5 human TM cells were seeded into 6-well plates the day before infection and reached 70–80% confluence the next day. Human TM cells were infected with Empty, MYOC-WT or MYOC-N450Y lentivirus at a multiplicity of infection of 5, and polybrene was added to the cells at a concentration of 5 µg/ml. After 20 h of infection, the culture medium was replaced with fresh medium. Human TM cells were harvested for subsequent analysis at indicated timepoints.

### Adenovirus Infection

48 hours after infection with lentivirus, GFP-LC3B adenovirus was added to fresh culture medium of human TM cells. GFP fluorescence was captured using ZEISS microscopy. GFP-LC3 adenovirus was purchased from WZ Biosciences.

### Drug Treatment

Stock solution of Dexamethasone (100 mM), Rapamycin (100 mM) and Chloroquine (100 mM) were made using dimethyl sulfoxide. All the drugs were diluted in culture medium for human TM cell treatment. The concentrations used in this study were 100 nM for Dexamethasone, various concentrations (20, 30, 50 μM) for Rapamycin and 100 μM for Chloroquine. Dimethyl sulfoxide was used as control.

### Western Blot Analysis

Radioimmunoprecipitation buffer (Thermo, Waltham, United States) was used for protein extraction. 40 μg total proteins, 10 μL supernatant or 4 µl aqueous humor were separated using sodium dodecyl sulfate-polyacrylamide gel electrophoresis (SDS-PAGE) and transferred to polyvinylidene difluoride membranes (Thermo). The membrane was then blocked in nonfat milk (Thermo) for 2 h. After incubation with primary antibodies at 4°C overnight and with secondary antibodies at room temperature for 2 h, the membranes were washed with 0.1% tris-buffered saline with Tween and subjected to chemiluminescence analysis using the enhanced chemiluminescent reagent kit (CWBIO, Beijing, China). The antibodies against myocilin (sc-515500, sc-137233) and GAPDH (sc-25778) were purchased from Santa Cruz biotechnology (Dallas, TX, United States). Antibodies against Grp78 (3177), Grp94 (2104), p62 (5114), LC3B (43566), mTOR (2983) and p-mTOR (2974) were purchased from Cell Signaling Technology (Boston, MA, United States). All the secondary antibodies, anti-Mouse IgG (7076) and anti-Rabbit IgG (7074), were purchased from Cell Signaling Technology.

### Immunostaining

Human TM cells and sectioned tissues were washed with PBS, fixed with 4% paraformaldehyde (CWBIO) for 12 min, permeabilized with 0.3% TritonX-100 (CWBIO) and blocked with 5% albumin serum (BSA) for 1 h. The samples were then incubated with primary antibodies at 4°C overnight, followed by 1-h incubation with Alexa Fluor 488 (A21206; Thermo) or 546 (A10036; Thermo) secondary antibodies. Nuclei were stained with 4’, 6-diamidino-2-phenylindole (Vector Laboratories, Peterborough, United Kingdom). Images were captured with a Zeiss or Leica confocal imaging system (Carl Zeiss; Hertfordshire, United Kingdom). Primary antibody myocilin (sc-515500, Santa Cruz), RBPMS(GTX118619, GeneTex) and KDEL (ab176333, abcam) were diluted at a concentration of 1:200.

### Cell Viability Detection

Cells were seeded into 96-well plates at a density of 1,000 cells/well and 10 μL of CCK-8 solution was added to each well every day at the same time for four days. After CCK8 addition, the cells were maintained at 37°C for 2 h, then spectrometric absorbance at 450 nm was detected on the microplate reader (Thermo).

### Apoptosis Assay

Cell apoptosis was measured using an Annexin V-FITC/PI apoptosis detection kit (CWBIO) according to the manufacturer’s protocol. In brief, cells were washed three times using PBS (Gibco) and trypsinized with 0.05% trypsin (Thermo). The cells were then harvested and resuspended in 250 µl of binding buffer to reach a concentration of 1.0 × 10^6/ml. 5 μL of annexin V-FITC and 10 μL of PI were added to 100 μL cell suspension. The mixture was incubated for 15 min at room temperature, and then subjected to flow cytometry detection.

### Construction of Transgenic Mice

This study was approved and monitored by the Institutional Animal Care and Use Committee of the Capital Medical University of Beijing (IACUC; AEEI-2018-198), and conformed to the National Institute of Health Guide for the Care and Use of Laboratory Animals as well as the Association for Research in Vision and Ophthalmology Statement for the Use of Animals in Ophthalmic and Vision Research.

The cDNA containing Asn 450 Tyr mutation in WT MYOC gene was introduced using the Quick Change Site-Directed Mutagenesis kit (Stratagene) and cloned into donor vector. The CRISPR/Cas9 system was used to construct the transgenic mice by homologous recombination. Cas9 mRNA, gRNA and donor vector were injected into fertilized zygotes of C57BL/6 J mice. F0 lines were genotyped by polymerase chain reaction and Sanger sequencing. F0 lines were then mated with C57BL/6 J mice, and F3 and later generations were used in this study. The primers used for genotyping are specific to human MYOC, Forward: CGT​GCC​TAA​TGG​GAG​GTC​TAT; Reverse: CTG​GTC​CAA​GGT​CAA​TTG​GT.

### IOP Measurement

The animals were anesthetized with isoflurane and oxygen for 5 min. IOP was measured between 3:00 and 5:00 p.m. using rebound tonometry as described previously (McDowell et al., 2022). This involves gently pressing the measurement button and the probe tip located 1–4 mm from the corneal surface strikes the central cornea five times consecutively. The above procedure was repeated three times and the readings were averaged.

### Slit Lamp Examination

Slit-lamp (HAAG-STREIT AG, Swiss, BX-900) examination was used to evaluate the anterior segment, including iris, lens and cornea. Photographs were captured using a digital camera (Canon) using consistent parameters throughout.

### Gadolinium Magnetic Resonance Imaging

Gd-MRI was applied to analyze aqueous humor dynamics as previously described (Ho et al., 2014; Nair et al., 2021). All MRI analyses were performed using a Pharmascan 7T (BRUKER). Briefly, mice were anesthetized with 2% isoflurane and kept warm during the experimental process. Respiration rate was monitored using a small pneumatic pillow. Mice were placed in position to acquire a baseline measurement and were injected with Gadolinium-DTPA intraperitoneally at a dose of 0.3 mmol/kg. RARE-T1-weighted MRI was acquired every 10 min for 1 h. The imaging parameters were as follows: repetition time/echo time = 600/9 ms, field of view = 1.8 × 1.8 cm^2^, and in-plane resolution = 256 × 256 μm^2^. Seven 1-mm-thick slices were positioned across the globes in a coronal orientation using scout T2-weighted MR images in all coronal, transverse, and sagittal planes as references for reproducible localization.

### Hematoxylin and Eosin Staining

Enucleated eyes were washed with phosphate buffered saline (PBS) three times, fixed in 4% paraformaldehyde, dehydrated with gradient ethanol and embedded in paraffin. Tissues were sliced for 5 μm and stained with hematoxylin and eosin.

### Virtual Optokinetic System

Optokinetic responses were stimulated using sinusoids at a range of spatial frequencies to evaluate visual function as previously described (Prusky et al., 2004). The animals were placed on a platform to adapt for about 5 min. The grating speed was adjusted to 12°/s during the experiment, and the grating spatial frequency ranged from 0.2 to 0.6 cycles/degree (c/d) in a staircase manner. The experimenter selected “Yes” or “No” to indicate whether the animal tracks or does not track the stimulus. The duration of each experiment was 5–10 min, and the interval time for each animal between two adjacent experiments was at least 10 min.

### Statistical Analysis

Statistical data are reported as mean ± standard error of the mean (SEM) of at least three independent repeats biologically. Statistical analysis was performed using GraphPad Prism software. Student’s t-test was used to compare two groups and one-way analysis of variance (ANOVA) was used for more than two groups. A *p* value of less than 0.05 was considered significant.

## Results

### Non -Secretion of Mutant MYOC Induced ER Stress

**FIGURE 1:**
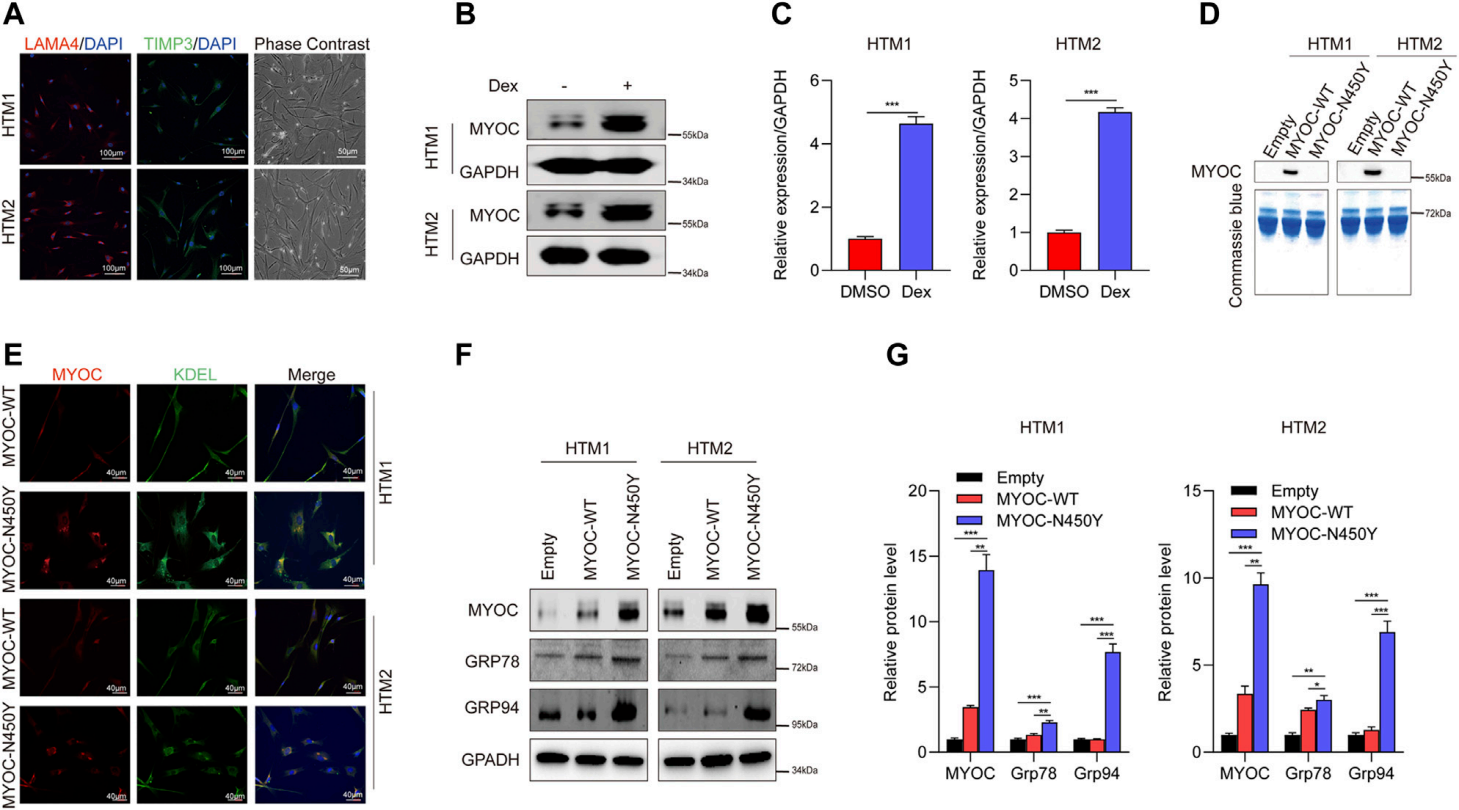
Non-secretion of mutant MYOC-induced ER stress. **(A)** Immunofluorescence staining of LAMA 4 (red) and TIMP3 (Green) and phase contrast images in HTM cells.**(B)** Western blot detection of MYOC and GAPDH with or without dexamethasone treatment. **(C)** Bands intensity analysis in **(B)** using ImageJ software. ***, *p* < 0.001. **(D)** Western blot analysis of MYOC in culture medium of HTM cells overexpressing MYOC-WT or MYOC-N450Y **(E)**. Immunofluorescence staining of MYOC (red) and KDEL (green) in HTM cells overexpressing MYOC-WT or MYOC-N450Y. **(F)** Western blot detection of MYOC, Grp78, Grp94 and GAPDH in HTM cells overexpressing MYOC-WT or MYOC-N450Y **(G)**. Band intensity analysis in **(F)** using ImageJ software. *, *p* < 0.05; **, *p* < 0.01; ***, *p* < 0.001. Empty: empty vector lentiviruses; MYOC-WT: lentiviruses expressing wild type myocilin gene; MYOC-N450Y: Lentiviruses expressing N450Y mutant myocilin gene.

Previously, we reported that aggregation of N450Y mutant MYOC (MYOC-N450Y) intracellularly induced ER stress and caused damage to cells in one strain of human primary TM (HTM) cells (Yan et al., 2020). In the present study, we used two strains of HTM cells purchased from ScienCell Corporation. First, we conducted characterization of HTM cells as previously indicated (Wang et al., 2021) and confirmed the ER stress phenotype. Phase contrast images were captured using ZEISS microscopy. Immunofluorescence staining demonstrated that two strains of HTM cells expressed TM biomarkers, including TIMP3 and LAMA4 (Figure 1A), and MYOC protein level was increased after dexamethasone treatment (Figures 1B,C). Consistent with our previous study(Yan et al., 2020), MYOC-N450Y failed to secrete into the medium (Figure 1D), accumulated in ER (Figure 1E) and upregulated protein level of ER stress related proteins Grp 78 and Grp 94 (Figures 1F,G). These results show that non secretion of MYOC-N450Y accumulates in ER and induces ER stress.

### N450Y Mutant MYOC Activates Autophagy

**FIGURE 2:**
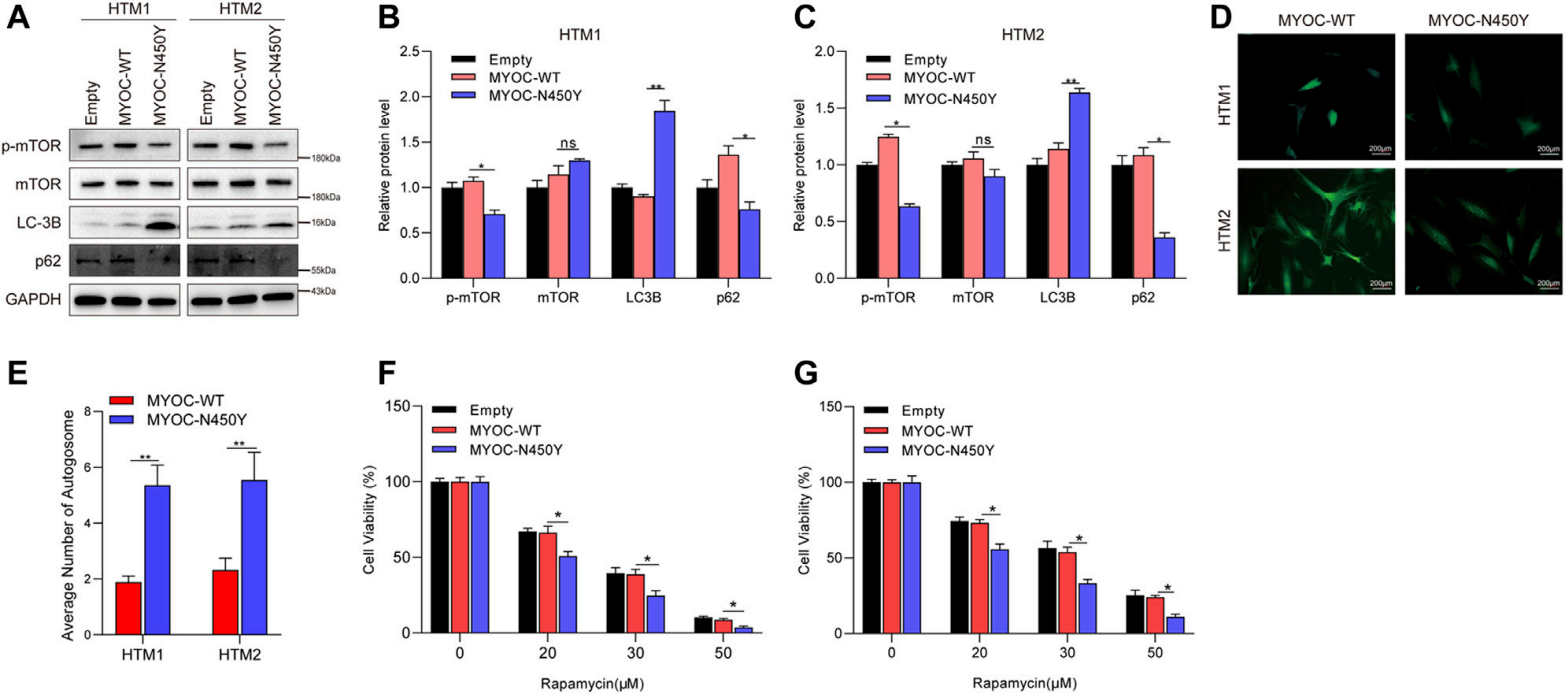
N450Y mutant MYOC activates autophagy. **(A)** Western blot detection of mTOR, p-mTOR, LC3B, P62 and GAPDH **(B,C)**. Band intensity analysis in **(A)** using ImageJ software. *, p < 0.05; **, p < 0.01; ns, no significance. **(D)** HTM cells were infected with lentiviruses expressing MYOC-WT or MYOC-N450Y for 48 h and transduced with EGFP-LC3 for 24 h **, p < 0.01. **(E)** Number of autophagosomes in **(D)**. **, p < 0.01. **(F,G)** Cell viability was detected using CCK8 after rapamycin treatment. *, *p* < 0.05. Empty: empty vector lentiviruses; MYOC-WT: lentiviruses expressing wild type myocilin gene; MYOC-N450Y: Lentiviruses expressing N450Y mutant myocilin gene.

To determine whether constant ER stress induced by N450Y mutant MYOC triggers autophagy, we analyzed the protein level of key autophagy related genes LC3B and p62 and autophagy regulator mTOR signaling. Synthesized LC3B is cleaved to form cytosolic LC3-I. When autophagy is induced, phosphatidylethanolamine is conjugated to LC3-I forming LC3-II. The ratio of LC3-Ⅱ/LC3-I or upregulation of LC3B is an indicator of autophagosome (Kuma et al., 2007; Meng et al., 2020). p62 as a ubiquitin receptor connected ubiquitinated cargos for autophagic degradation and decreased generally in autophagic process (Klionsky et al., 2012). The mTOR kinase is a key regulator of autophagy and activated mTOR signaling is reported to inhibit autophagy (Martina et al., 2012). As shown in Figures 2A–C, compared with the MYOC-WT group, MYOC-N450Y significantly increased protein level of LC3B and decreased the protein level of p62. GFP-LC3 is widely used to monitor the autophagosome. HTM cells were infected with lentivirus expressing either MYOC-WT or MYOC-N450Y for 48 h and transduced with Ad5 expressing GFP-LC3 again for 48 h. GFP puncta were observed by ZEISS microscopy and quantified. Compared with HTM cells expressing MYOC-WT, MYOC-N450Y demonstrated increased autophagosome (Figures 2D,E), indicating autophagy activation. Rapamycin, an inhibitor of mTOR signaling, is an inducer of autophagy. We examined the sensibility of HTM cells expressing either MYOC-WT or MYOC-N450Y and found that cells expressing MYOC-N450Y were more sensitive to rapamycin compared with the MYOC-WT group (Figures 2F,G). These data indicate that MYOC-N450Y activates autophagy in HTM cells.

### Autophagy Inhibition Rescues HTM Cells Viability and Apoptosis

**FIGURE 3:**
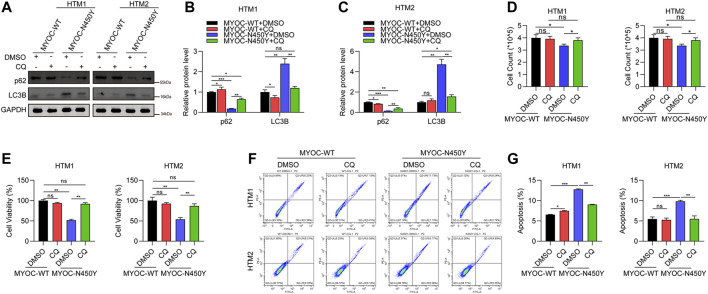
Autophagy inhibition rescues HTM cell viability and apoptosis **(A)** Western blot detection of P62, LC3B and GAPDH with or without chloroquine treatment. **(B,C)** Band intensity analysis in **(A)** using ImageJ software. *, *p* < 0.05; **, *p* < 0.01; ***, *p* < 0.001; ns, no significance. **(D)** Cell count of HTM cells with or without chloroquine treatment. **, *p* < 0.01. **(E)** Cell viability was detected using CCK8 in HTM cells with or without chloroquine treatment. **(F)** Flow cytometry analysis of apoptosis of HTM cells in different groups. **(G)** Apoptosis ratio analysis of HTM cells in different groups. ns, no significance; **, *p* < 0.01; ***, *p* < 0.001. Empty: empty vector lentiviruses; MYOC-WT: lentiviruses expressing wild type myocilin gene; MYOC-N450Y: Lentiviruses expressing N450Y mutant myocilin gene.

Chloroquine (CQ) is a commonly used autophagy inhibitor by targeting lysosome. To further verify the role of autophagy, HTM cells expressing either MYOC-WT or MYOC-N450Y were treated with CQ. HTM cells expressing MYOC-WT or MYOC-N450Y were treated with CQ for 12 h and autophagy related biomarkers were detected by Western Blot. p62 protein level was upregulated and LC3B protein level was decreased in HTM cells expressing MYOC-N450Y, compared to the MYOC-WT group (Figures 3A–C). An automatic cell counter and CCK8 were used to quantify cell numbers and viability respectively. As expected, cell numbers were evidently elevated (Figure 3D) and cell viability was rescued (Figure 3E) in MYOC-N450Y group compared with the MYOC-WT group. Then we determined whether autophagy inhibition alleviated cell death. Flow cytometry results indicated that ratio of cell death was reduced in HTM cells expressing MYOC-N450Y, compared with expressing MYOC-WT (Figures 3F,G). These data indicate that MYOC-N450Y impairs HTM cells by activating autophagy.

### Mutant Myocilin Protein Was Expressed in the Anterior Angle of Tg-MYOC^N450Y^ Mice

**FIGURE 4:**
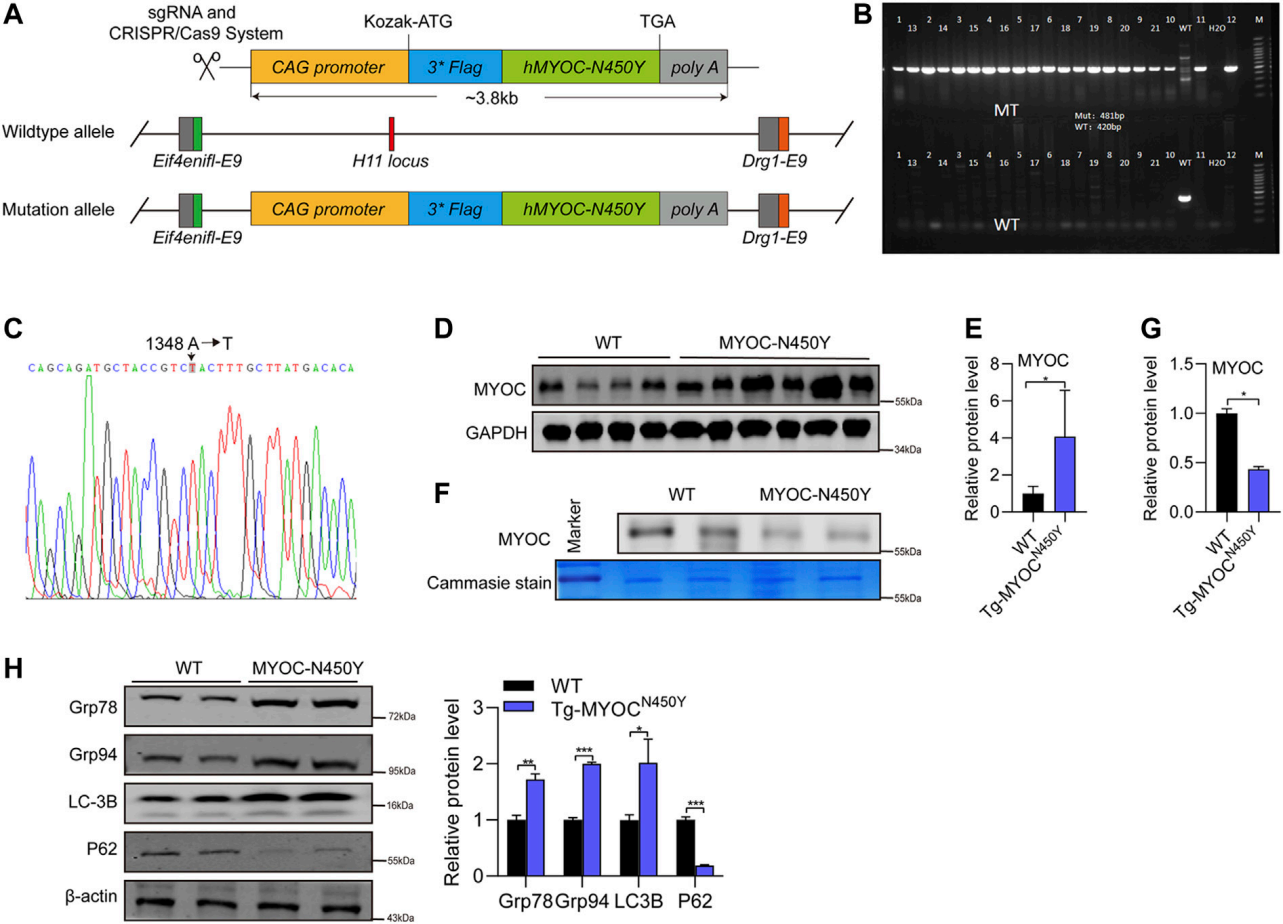
Mutant MYOC protein was expressed in the anterior angle of Tg-MYOC^N450Y^ mice **(A)** Schematic of Tg-MYOCN450Y construct. **(B)** PCR was applied to detect the expression of transgene MYOC-N450Y in Tg-MYOCN450Y mice at age of 5 months. **(C)** The presence of MYOC-N450Y was confirmed by sequencing. **(D)** Western blot was used to analyse the MYOC expression in anterior chamber angle tissue in WT littermates and Tg-MYOC^N450Y^ mice at 5 months of age. **(E)** Band intensity analysis in **(D)** using ImageJ software. *, p < 0.05. **(F)** Western blot was used to analysis MYOC secretion in aqueous humor. ns, no significance; *, p < 0.05. **(G)** Band intensity analysis in **(F)** using ImageJ software. *, p < 0.05. **(H)** Western blot was used to analyse the expression of Grp78, Grp94, LC3B, p62 and GAPDH at 8 months of age in WT littermates and Tg-MYOCN450Y mice.

To further determine whether MYOC-N450Y induces ER stress, activates autophagy and is associated with POAG *in vivo*, we constructed transgenic mice carrying human MYOC N450Y(c.A1348T) mutation (Tg-MYOC^N450Y^), illustrated by the schematic diagram in Figure 4A. Primers specific for mouse or human MYOC gene were designed to amplify the genome DNA of WT mice (C57BL/6 J) or Tg-MYOC^N450Y^. As shown in Figures 4B,C, human N450Y mutant MYOC gene was successfully inserted into the genome in mice, and N450Y mutant MYOC protein was over-expressed in the anterior angle tissue (Figures 4D,E). Consistent with our *in vitro* study, N450Y mutant MYOC protein failed to secrete into aqueous humor (Figures 4F,G), induced ER stress and stimulated autophagy *in vivo* (Figure 4H). These data demonstrate that Tg-MYOC^N450Y^ mice were successfully developed and MYOC-N450Y non-secretion induces ER stress and activates autophagy *in vivo*.

### Tg-MYOC^N450Y^ Mice Exhibit Glaucoma Phenotypes

**FIGURE 5:**
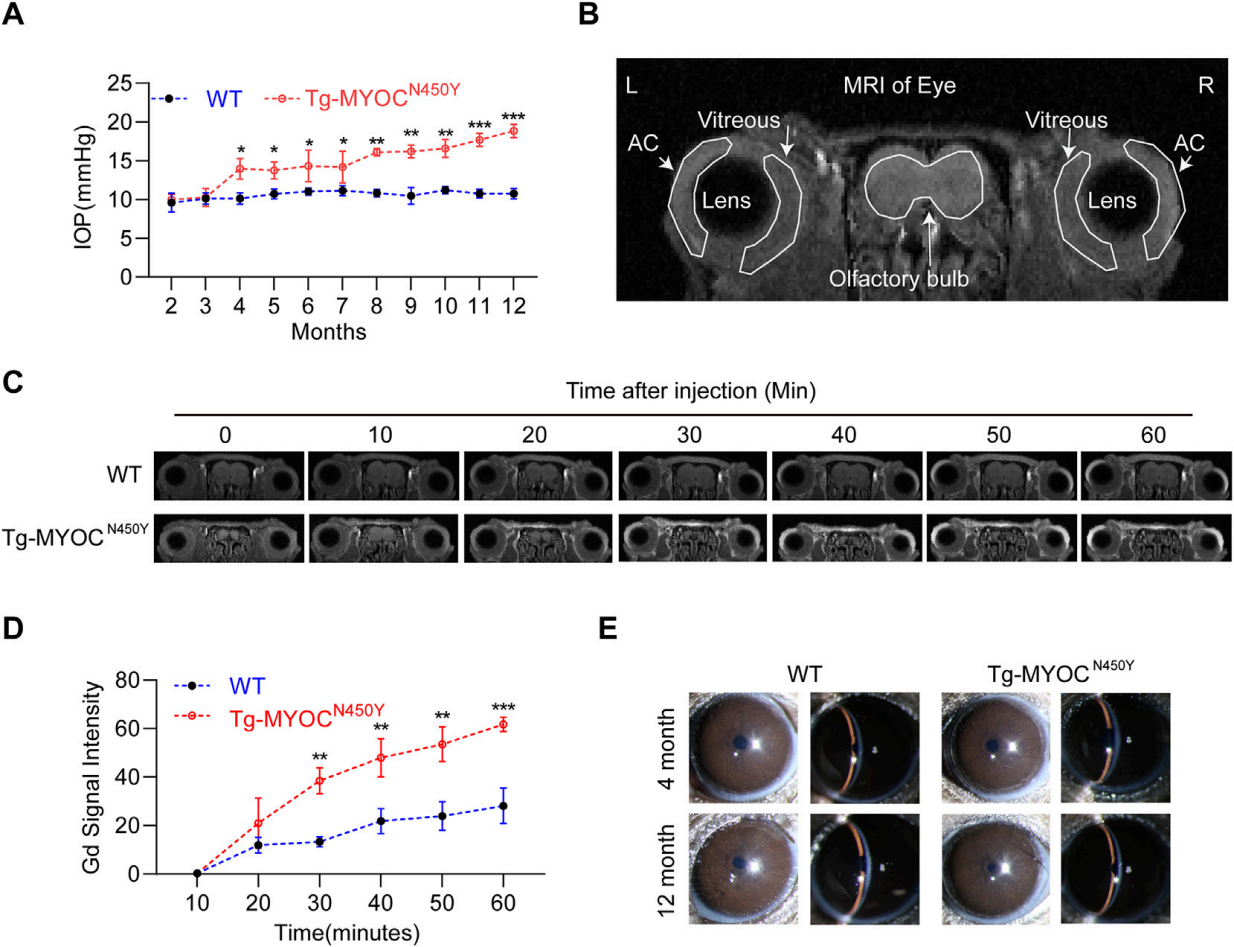
Mutant MYOC protein leads to IOP elevation and altered aqueous humor dynamics **(A)** IOP was measured in Tg-MYOC^N450Y^ and WT littermates at various ages. *, *p* < 0.05; **, *p* < 0.01; ***, *p* < 0.001. **(B)** Representative MR image of basic mice eye anatomy. AC, anterior chamber. **(C)** Gd-enhanced MR images from WT littermates and Tg-MYOC^N450Y^ mice at age of 8 months. **(D)** Pixel intensity was evaluated by Image J software in AC angle after Gd-DTPA injection. **, *p* < 0.01; ***, *p* < 0.001. **(E)** Slit lamp examination of WT and Tg-MYOC^N450Y^ mice at various ages.

MYOC-N450Y has been identified in the pedigrees of POAG patients and is correlated with elevated IOP (Zhao et al., 2010). Figure 5A shows that IOP of Tg-MYOC^N450Y^ mice was elevated compared with WT mice at 4 months and remained high until 12 months of age, the last experimental time point. To determine whether elevated IOP was associated with aqueous humor outflow impedance, we performed Gadolinium magnetic resonance imaging (Gd-MRI) on 8 months of WT littermates and Tg-MYOC^N450Y^ mice. Representative MR image and basic mouse eye anatomy are shown in Figure 5B. Analysis of MRI results showed that, compared with WT mice, aqueous humor outflow was significantly decreased in Tg-MYOC^N450Y^ mice (Figures 5C,D) tracked by the accumulated Gd signal in the ocular anterior segment. Our results suggested that Gd signal accumulation was significantly increased after 30 min of scanning and remained raised until the end of the test. Moreover, no significant change was observed between WT and Tg-MYOC^N450Y^ mice in the cornea or anterior chamber at 4 months or 12 months of age (Figure 5E).

**FIGURE 6:**
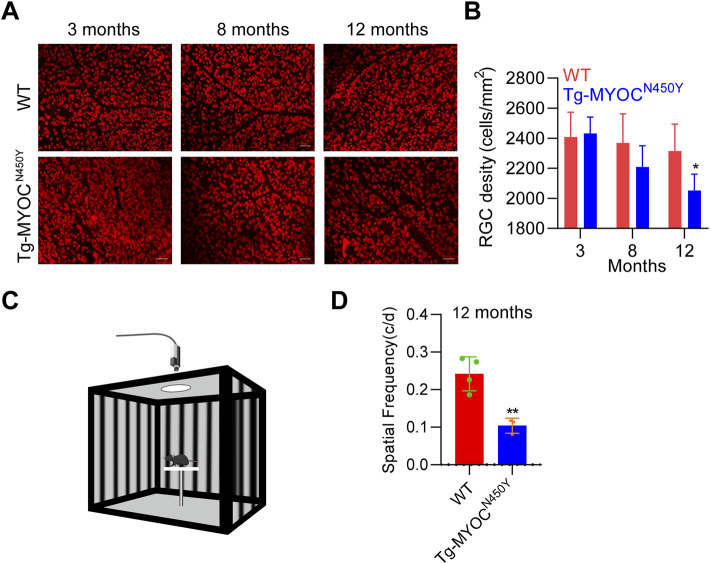
Mutant MYOC leads to RGC loss and visual impairment **(A)** Whole mount retinal preparations were stained with RBPMS antibody in WT littermates and Tg-MYOC^N450Y^ mice at 3, 8 and 12 months of age. **(B)** Average RGC numbers of **(A)** were quantified using ImageJ software. *, *p* < 0.05. **(C)** Diagram of virtual optokinetic system. **(D)** The virtual optokinetic system was applied to evaluate the maximum spatial frequency in WT littermates and Tg-MYOC^N450Y^ mice at age of 12 months. **, *p* < 0.01.

To further explore whether constant IOP elevation leads to RGC loss, we performed retinal flat mount immunostaining of RBPMS biomarker of RGC, and demonstrated that the density of RGCs was markedly decreased in Tg-MYOC^N450Y^ mice at 12 months of age, whereas no difference was found at either 3 months or 8 months of age, compared with WT littermates (Figures 6A,B). Generally, RGC loss ultimately leads to impaired vision. We evaluated visual function by measuring optomotor head-tracking responses to a grating rotating at 12 degrees per second (see diagram in Figure 6C). As shown in Figure 6D, the acuity was significantly decreased in Tg-MYOC^N450Y^ mice compared with WT mice. These results indicate that Tg-MYOC^N450Y^ mice exhibit glaucoma phenotypes, with aqueous humor outflow resistance.

## Discussion

Dysfunction of TM cells is the main cause of IOP elevation in glaucoma but the mechanism underpinning TM damage is unclear. MYOC gene mutations leads to glaucoma mainly through TM cell death inducing IOP elevation. In our previous study, MYOC-N450Y aggregates at cells, induced ER stress and promotes apoptosis in TM cells (Yan et al., 2020). Dysfunction of TM cells is the main cause of IOP elevation in glaucoma but the mechanism underpinning TM damage is unclear. MYOC gene mutations leads to glaucoma mainly through TM cell death inducing IOP elevation. In our previous study, MYOC-N450Y aggregates at cells, induced ER stress and promotes apoptosis in TM cells.

In our study, RGC loss occurred later than 8 months although IOP elevation was evident in 4 months old Tg-MYOC^N450Y^ mice. The finding that RGC loss lags a few months behind IOP elevation is consistent with a previous study(Park et al., 2012). We reason that IOP elevation is low in the first 8 months of age in Tg-MYOC^N450Y^ mice. While sustained IOP elevation leads to TM dysfunction, the degree of IOP elevation is not sufficient to cause RGC loss and RGC is resistant to low level pressure at early and middle age in Tg-MYOC^N450Y^ mice. However, at 12 months of age, RGCs are more sensitive to pressure and greatly increased IOP dramatically impairs RGC survival in Tg-MYOC^N450Y^ mice.

In response to ER stress, autophagy may be cytoprotective by degrading aggregated misfolded proteins and recycling the materials for cellular use (Ogata et al., 2006; Yorimitsu et al., 2006). But in some circumstances, autophagy may be detrimental. In gastric cancer cells, ER stress-induced autophagy promotes cell death under hypoxia rather than normoxia (Kim et al., 2020). In the context of amino acid starvation, autophagy promotes cell death of mouse embryonic fibroblasts (Gao et al., 2015), and in autosomal dominant optic atrophy, inhibition of autophagy curtails visual loss (Zaninello et al., 2020). Consistent with this, in a chronic hypertensive glaucoma model, autophagy stimulation induces RGC death (Zaninello et al., 2020). In our study, N450Y mutant MYOC activated autophagy as indicated by increased LC3B and decreased p62. Stimulated autophagy plays a harmful role in TM cells as evidenced by inhibition of autophagy rescuing cell viability and apoptosis in TM cells expressing MYOC-N450Y. In another study, however, Y437H mutant MYOC leads to autophagy dysfunction thus impairing TM cells, whereas inhibition of autophagy aggravates mutant MYOC aggregation and worsens IOP elevation *in vivo* (Kasetti et al., 2021). We proposed that alternative mechanisms may exist based on different sites of MYOC mutation. Previous studies showed that the half-lives of WT, P370L mutant and Q368X mutant MYOC were 24 h, 42 and 50 h respectively indicating different cellular processing (Qiu et al., 2014). It is suggested that the proteolytic cleavage degree in linker domain of mutant MYOC is correlated with severity of glaucoma (Wang et al., 2019). Pro 370 Leu, involved in the most severe glaucoma phenotype, exhibits a high degree of proteolytic cleavage inhibition (Wei et al., 2011). whereas in Glu 323 Lys and Asp 380 Ala mutation sites, related to less severe glaucoma, proteolytic cleavage inhibition is less severe (Aroca-Aguilar et al., 2005). The above studies support the present proposal.

In conclusion, our data demonstrate that MYOC-N450Y induces ER stress, activates autophagy and leads to glaucoma phenotypes *in vivo*. Activation of autophagy exacerbates TM cells viability, whereas inhibition of autophagy rescues TM cell viability and apoptosis. These results indicate the association between ER stress and autophagy in TM dysfunction and novel strategies targeting autophagy may be developed in N450Y mutant MYOC related to POAG.

## Data Availability

The original contributions presented in the study are included in the article/Supplementary Material, further inquiries can be directed to the corresponding authors.
